# Targeting Lactylation: From Metabolic Reprogramming to Precision Therapeutics in Liver Diseases

**DOI:** 10.3390/biom15081178

**Published:** 2025-08-16

**Authors:** Qinghai Tan, Mei Liu, Xiang Tao

**Affiliations:** Department of Gastroenterology, Tongji Hospital, Tongji Medical College, Huazhong University of Science and Technology, Wuhan 430030, China

**Keywords:** lactylation, lactate, post-translational modification, liver diseases, metabolic regulation

## Abstract

Lactylation, a recently identified post-translational modification (PTM) triggered by excessive lactate accumulation, has emerged as a crucial regulator linking metabolic reprogramming to pathological processes in liver diseases. In hepatic contexts, aberrant lactylation contributes to a range of pathological processes, including inflammation, dysregulation of lipid metabolism, angiogenesis, and fibrosis. Importantly, lactylation has been shown to impact tumor growth, metastasis, and therapy resistance by modulating oncogene expression, metabolic adaptation, stemness, angiogenesis, and altering the tumor microenvironment (TME). This review synthesizes current knowledge on the biochemical mechanisms of lactylation, encompassing both enzymatic and non-enzymatic pathways, and its roles in specific liver diseases. From a therapeutic perspective, targeting lactate availability and transport, as well as the enzymes regulating lactylation, has demonstrated promise in preclinical models. Additionally, combinatorial approaches and natural compounds have shown efficacy in disrupting lactylation-driven pathways, providing insights into future research directions for hepatic diseases. Although the emerging role of lactylation is gaining attention, its spatiotemporal dynamics and potential for clinical translation are not yet well comprehended. This review aims to synthesize the multifaceted roles of lactylation, thereby bridging mechanistic insights with actionable therapeutic strategies for liver diseases.

## 1. Introduction

The Warburg effect, characterized by aerobic glycolysis and lactate accumulation, has been long established as a metabolic hallmark of cancer and inflammatory diseases. Traditionally considered merely a metabolic byproduct, lactate has recently been identified as a dynamic signaling molecule in emerging studies. This paradigm shift is largely attributed to the discovery of lysine lactylation (Kla), a novel post-translational modification (PTM) that establishes a direct link between glycolytic flux and both epigenetic and non-epigenetic regulatory networks [1,2,3,4]. Lactylation modification, characterized by the addition of lactyl groups to lysine residues, occurs on both histone and non-histone proteins, thereby exerting a broad influence on gene expression and cellular functions [4,5]. This process is regulated by a confluence of metabolic activity, enzymatic action, and environmental conditions. Glycolysis and its byproduct lactate serve as central drivers of lactylation, while specific enzymes modulate its addition and removal [6]. This reversible nature of lactylation makes it a potential therapeutic target across a wide range of diseases, especially in cancers [7,8,9].

The liver plays a pivotal role in maintaining metabolic homeostasis, detoxification, and the synthesis of vital proteins, making it susceptible to a range of pathologies, particularly metabolic disorders. Liver diseases encompass a wide range of conditions that affect the liver’s ability to function properly, leading to significant morbidity and mortality worldwide. The significant burden of these diseases highlights the necessity of quantifying their impact and formulating strategies to alleviate their effects on public health [10,11,12]. Recent evidence indicates that dysregulated lactylation contributes to the progression of liver diseases [13,14,15]. Furthermore, targeting lactate transporters and critical enzymes involved in lactylation offers promising avenues for novel liver diseases therapies [3,13,16,17]. Additionally, by targeting lactylation and leveraging combination strategies, there is potential to overcome immunotherapy resistance and improve patient outcomes in various cancer types [18,19,20,21]. These insights collectively underscore the importance of a holistic approach in the development of next-generation liver disease therapies.

Despite recent advancements, several critical knowledge gaps remain. This review systematically synthesizes current knowledge regarding the biochemical mechanisms of lactylation, including both enzymatic and non-enzymatic pathways, and its roles in specific liver diseases. Additionally, we systematically catalog compounds that influence lactate availability, transport, and lactylation, thus offering a comprehensive framework for lactylation-targeted therapies. By synthesizing mechanistic insights with translational perspectives, our objective is to bridge the existing knowledge gap regarding the role of lactylation in these diseases.

## 2. Biochemical Mechanisms of Lactylation

Lactate, a significant carbon metabolite, has been the subject of extensive research due to its association with the Warburg effect, a process where cancer cells prefer glycolysis instead of oxidative phosphorylation even when there is enough oxygen [22,23]. In the glycolytic pathway, glucose is converted into two pyruvate molecules, which are later transformed into lactate by the enzyme lactate dehydrogenase A (LDHA) [24]. Lactate is subsequently converted into lactyl-CoA, facilitated by enzymes such as acetyl-CoA synthetase 2 (ACSS2) and guanosine triphosphate-specific succinyl-CoA synthetase (GTPSCS). ACSS2, traditionally associated with acetyl-CoA synthesis, facilitates histone lactylation via lysine acetyltransferase 2A (KAT2A), also known as general control non-depressible 5 (GCN5), to promote tumor immune evasion [25], while GTPSCS drives lactyl-CoA production to support gliomagenesis [26]. Lactyl-CoA serves as the substrate for lactylation, a modification mediated by “writer” enzymes, including histone acetyltransferases (HATs) such as p300/CBP, GCN5, KAT5, KAT7 (HBO1), and KAT8 [27,28,29,30,31,32], as well as other modifiers like α-tubulin acetyltransferase 1 (ATAT1) [33]. Histone deacetylase 6 (HDAC6) is also categorized here [34], potentially reflecting its dual role in acetylation or lactylation dynamics. The reversal of lactylation by “erasers” involves classical deacetylases, including histone deacetylases (HDACs) HDAC1-3 and sirtuins1-3 (SIRT1-3), suggesting mechanistic overlap between acetylation and lactylation removal [35]. In Escherichia coli, lactate is converted to lactyl-CoA via YdiF. Lactyl-CoA then serves as a substrate for lactylation modifications, potentially mediated by YiaC, a speculated acetyltransferase-like enzyme. The removal of lactylation is suggested to involve CobB, an NAD^+^-dependent deacetylase homologous to sirtuins, which hydrolyzes lactylated residues and releases nicotinamide (NAM) [36]. Notably, recent studies discovered alanyl-tRNA synthetase 1 (AARS1), its mitochondrial isozyme AARS2, and their Escherichia coli orthologue AlaRS as intracellular lactate sensors and lactyltransferases. In the presence of ATP, they could identify lactate and form lactate-AMP, releasing pyrophosphate (PPi). Then, the lactyl group is transferred to lysine residues on the substrate by AARS1/2 or AlaRS, completing lactylation and releasing AMP. This process involves direct lactylation and bypasses the need for lactyl-CoA [24,37,38,39]. Notably, recent studies identify Brg1 and DPF2 as “readers” of histone lactylation. The concurrent enrichment of both H3K18 lactylation (H3K18la) and these “readers” at the promoters of genes indicates a coordinated mechanism through which lactylation modulates gene transcription and expression, thereby enhancing the current understanding of histone modifications [40,41].

**Figure 1:**
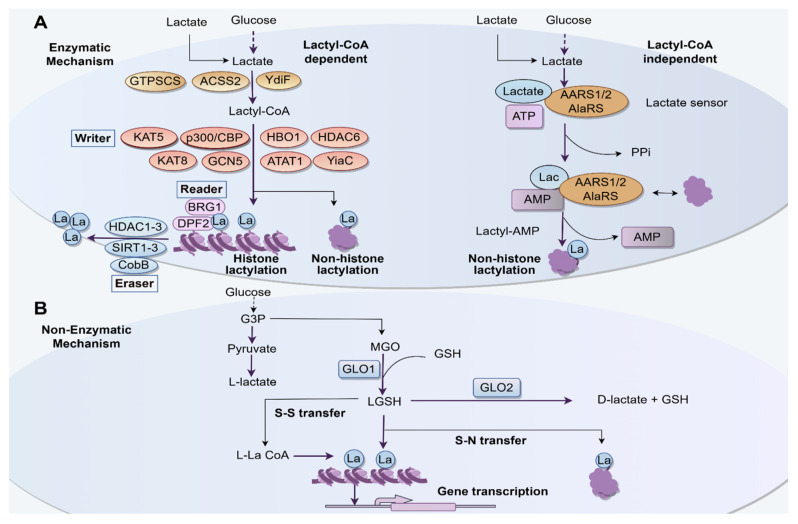
Schematic overview of enzymatic and non-enzymatic lactylation mechanisms linked to glycolysis and epigenetic regulation. (**A**) Enzymatic lactylation: this diagram outlines two proposed pathways for protein lactylation, lactyl-CoA-dependent and lactyl-CoA-independent mechanisms, alongside associated enzymatic “writers”, “erasers”, and “readers”. (**B**) Non-enzymatic lactylation: lactyl-CoA or reactive metabolites LGSH drive spontaneous lactylation of histones and non-histone proteins, influencing gene transcription and cellular signaling. AARS1/2: alanyl-tRNA synthetase 1/2; ACSS2, acetyl-CoA synthetase 2; ATAT1, α-tubulin acetyl-transferase 1; G3P, glyceraldehyde-3-phosphate; GCN5, general control non-depressible 5; GLO1, glyoxalase 1; GLO2, glyoxalase 2; GTPSCS, guanosine triphosphate-specific succinyl-CoA synthetase; HDAC, histone deacetylase; KAT, lysine acetyltransferase; LGSH, lactoylglutathione; L-La CoA, L-lactyl-CoA; MGO, methylglyoxal.

In addition, studies identified non-enzymatic acyl transfer of the lactate moiety from lactoylglutathione (LGSH) to protein lysine residues, generating a lactylation modification on proteins. Glucose undergoes glycolysis to yield glyceraldehyde-3-phosphate (G3P) and subsequently pyruvate. Under anaerobic conditions, pyruvate is reduced to L-lactate, a classical end-product of glycolysis. Parallel to this, methylglyoxal (MGO), a reactive dicarbonyl byproduct of glycolysis, is detoxified via the glyoxalase system. Glyoxalase 1 (GLO1) catalyzes the conjugation of MGO with reduced glutathione (GSH) to form LGSH, which is further hydrolyzed by glyoxalase 2 (GLO2) into D-lactate and regenerated GSH. LGSH can directly donate lactyl groups to target proteins for chemical lactylation. Notably, L-lactate is further metabolized to L-lactyl-CoA (L-La CoA), a potential acyl-donor for non-enzymatic lactylation. This process facilitates the covalent attachment of lactate groups to histones or non-histone proteins, independent of enzymatic catalysis (Figure 1). These lactylation events are implicated in regulating gene transcription, potentially mediating cellular adaptation to metabolic states, such as hypoxia or nutrient stress [42,43,44]. Ultimately, lactylation modulates gene expression, linking metabolic states to epigenetic regulation [5,45,46,47].

## 3. Crosstalk with Other Protein Modifications

**Figure 2:**
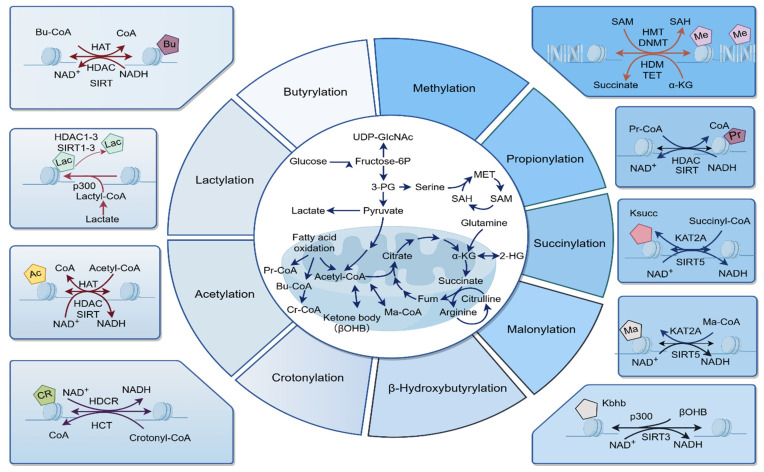
Crosstalk between lactylation and other PTMs. This schematic illustrates the complex interplay between Kla and other key PTMs (e.g., acetylation, methylation, crotonylation, succinylation, β-hydroxybutyrylation, butyrylation, propionylation, and malonylation). Specifically, lactylation engages in intricate, bidirectional crosstalk with major PTM networks through mechanisms such as direct competition for lysine residues, shared enzymatic pathways, interdependence and competition of metabolic precursors, and functional synergy or antagonism affecting chromatin structure and transcriptional regulation. DNMT, DNA methyltransferase; HAT, histone acetyltransferase; HCT, histone crotonyltransferase; HDAC, histone deacetylase; HDCR, histone decrotonylation; HDM, histone demethylase; HMT, histone methyltransferase; KAT2A, lysine acetyltransferase 2A; SAH, S-adenosylmethionine; SAM, S-adenosylmethionine; SIRT, sirtuin; TET, ten-eleven translocation.

The metabolism of glucose, fatty acids, and amino acids is essential for cellular energy production and biosynthesis, resulting in a wide array of intermediate products that are crucial to cellular processes. Glucose metabolism, primarily through glycolysis and the tricarboxylic acid (TCA) cycle, produces pyruvate and lactate, which are key intermediates in energy production and biosynthetic pathways. Under aerobic conditions, glucose undergoes complete oxidation in the TCA cycle; however, under anaerobic conditions, it can be converted to lactate, which serves as a significant carbon source for the TCA cycle in various tissues and tumors [48]. In contrast, fatty acids are predominantly oxidized in the mitochondria to generate acetyl-CoA, which then enters the TCA cycle. Fatty acids can also produce other products, such as butyryl-CoA, crotonyl-CoA, and propionyl-CoA, which provide acyl groups to proteins. Furthermore, amino acids can be metabolized into intermediates like succinyl-CoA, which participates in the TCA cycle and other metabolic pathways [49,50] (Figure 2). These studies suggest a bidirectional interaction within PTM networks and among metabolic pathways, facilitating metabolic regulation.

Protein modifications are crucial in regulating a wide range of cellular processes and are involved in numerous physiological and pathological contexts. Among the most common types of protein modifications are acetylation, ubiquitination, methylation, SUMOylation, glycosylation, butyrylation, succinylation, crotonylation, propionylation, malonylation, and phosphorylation, each of which uniquely contributes to cellular function and regulation [14,50,51,52]. Kla, driven by lactate, exhibits structural similarity to lysine acetylation, which has been extensively studied for its involvement in essential cellular processes modulating protein stability, enzymatic activity, subcellular localization, and by regulating protein–protein and protein–DNA interactions [53]. The competition between lactylation and acetylation on lysine residues is a key research area, indicating complex interactions affecting metabolism and gene regulation. Enzymes like SIRT1 and SIRT3, identified as lysine delactylases, add complexity to these interactions [54]. Notably, pyruvate dehydrogenase complex (PDC) was at the epigenetic crossroads of lactylation and of acetylation. In the context of cancer, acetylation of pyruvate dehydrogenase complex component X (PDHX) has been demonstrated to disrupt the assembly of PDC, resulting in elevated lactate production and subsequent lactylation-mediated gene expression, thereby facilitating tumor progression. This underscores the potential regulatory interplay between acetylation and lactylation [55,56]. Furthermore, lactate has been shown to promote the lactylation and acetylation of high mobility group box-1 (HMGB1) in macrophages during polymicrobial sepsis, suggesting a synergistic role of lactylation and acetylation in modulating immune responses and inflammation [57]. Similarly, the Glis1-enhanced glycolytic flux increases cellular acetyl-CoA and lactate levels, subsequently enhancing acetylation (H3K27Ac) and lactylation (H3K18la), thereby facilitating cellular processes [58], unveiling a complex signaling cascade that involves the coordination of histone acetylation and lactylation driven by glycolysis.

In addition to acetylation, lactylation has also been associated with the regulation of gene expression through its impact on histone methylation, which is essential for chromatin structure and function, especially on lysine and arginine residues [59,60,61]. For example, histone lactylation has been implicated in the regulation of early embryonic development via its interaction with N6-methyladenosine (m6A) RNA methylation, particularly involving the m6A methyltransferase methyltransferase-like 3 (METTL3) [62]. In the context of disease, the modulation of METTL3 by histone lactylation has been demonstrated to promote ossification of the ligamentum flavum by enhancing the m6A methylation of bone morphogenetic protein 2 (BMP2) [63]. In another study, lactylation of ATP-binding cassette subfamily F member 1 (ABCF1) at the K430 residue facilitates its translocation into the nucleus, where it binds to the promoter of lysine demethylase 3A (KDM3A). This interaction upregulates KDM3A expression, subsequently activating the KDM3A-H3K9me2-hypoxia-inducible factor 1A (HIF1A) axis. Moreover, lactylation on specific lysine residues co-localizes with other PTMs, suggesting potential crosstalk among these modifications [64].

In a similar manner, lactylation may utilize the same enzymes or lysine residues as other PTMs, including crotonylation, succinylation, β-hydroxybutyrylation, butyrylation, propionylation, and malonylation [50,65,66] (Figure 2). Consequently, lactylation engages in intricate, bidirectional crosstalk with major PTM networks through mechanisms such as direct competition for lysine residues, shared enzymatic pathways, interdependence and competition of metabolic precursors, and functional synergy or antagonism affecting chromatin structure and transcriptional regulation. Elucidating this interplay is essential for the development of innovative therapeutic strategies targeting these PTMs.

## 4. Lactylation in Liver Diseases

### 4.1. Metabolic Dysfunction-Associated Steatotic Liver Disease (MASLD)

MASLD, previously known as non-alcoholic fatty liver disease (NAFLD), is characterized by hepatic fat accumulation in the presence of metabolic dysfunction without significant alcohol consumption. It is the leading cause of chronic liver disease in Western countries and is linked to metabolic syndrome, obesity, type 2 diabetes, and dyslipidemia. Globally, MASLD affects about 25% of the population, with prevalence varying by region and population [67,68,69,70,71]. It includes liver conditions from simple fat accumulation without inflammation to metabolic dysfunction-associated steatohepatitis (MASH), which can lead to fibrosis, cirrhosis, and liver cancer. There is no approved drug treatment for MASLD and management relies on lifestyle changes like weight loss, diet, and exercise [72,73].

**Figure 3:**
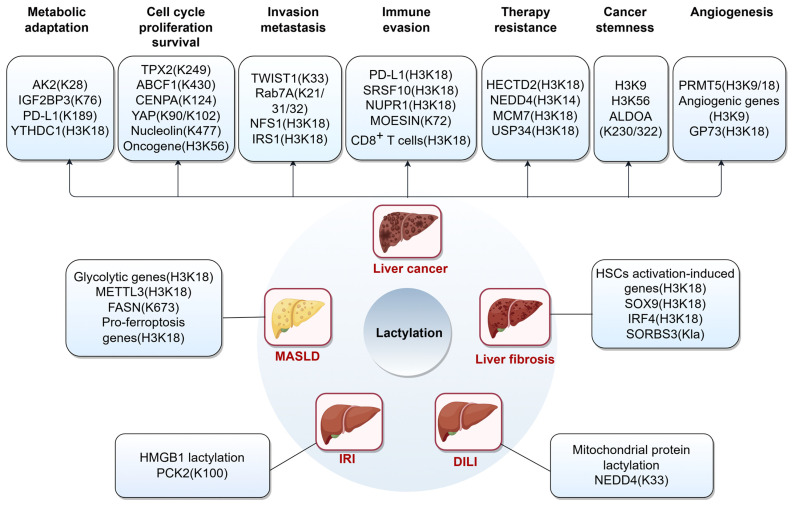
Schematic overview of non-histone protein lactylation targets and histone lactylation-associated pathways in liver diseases. This figure summarizes key protein targets undergoing Kla and histone lactylation-associated functional pathways across major hepatic pathologies. ABCF1, ATP-binding cassette subfamily F member 1; ALDOA, aldolase A; CENPA, centromere protein A; DILI, drug-induced liver injury; FASN, fatty acid synthase; GP73, Golgi phosphoprotein 73; H3K18, H3 lysine 18; HECTD2, HECT domain E3 ubiquitin protein ligase 2; HMGB1, high mobility group box-1; HSCs, hepatic stellate cells; IGF2BP2, insulin-like growth factor 2 mRNA binding protein 2; IRF4, interferon-regulatory factor 4; IRI, ischemia/reperfusion injury; IRS1, insulin receptor substrate 1; Kla, lysine lactylation; MASLD, metabolic dysfunction-associated steatotic liver disease; MCM7, minichromosome maintenance complex component 7; METTL3, methyltransferase-like 3; NEDD4, neuronal precursor cell-expressed developmentally downregulated 4; NFS1, cysteine desulfurase; NUPR1, nuclear protein 1; PCK2, phosphoenolpyruvate carboxykinase 2; PD-L1, programmed death-ligand 1; PRMT5, protein arginine methyltransferase 5; SORBS3, SH3 domain-containing 3; SRSF10, serine- and arginine-rich splicing factor 10; TPX2, targeting protein for Xklp2; TWIST1, twist family BHLH transcription factor 1; USP34, ubiquitin-specific protease 34; YAP, yes-associated protein.

Recently, lactylation has been identified as a significant player in the pathogenesis of MASLD (Figure 3), closely linked to the elevated lactate levels common in metabolic disorders like MASLD [74]. For example, it has been demonstrated that hexokinase 2 (HK2)-induced metabolic stress and inflammation in hepatic macrophages are intensified by histone lactylation, particularly at the H3K18 site. This lactate-dependent modification facilitates glycolysis and the M1 polarization of liver macrophages, establishing a feedback loop that exacerbates metabolic dysregulation and inflammation, thereby contributing to the pathogenesis of MASLD [75]. Moreover, the involvement of lactylation in MASLD is intricately linked to its effects on lipid metabolism. Specifically, histone lactylation at H3K18 and lactylation of fatty acid synthase (FASN) has been reported to facilitate hepatic lipid accumulation, suggesting that targeting lactylation pathways may represent a promising therapeutic strategy for MASLD management [76,77]. Recent investigations have elucidated the mechanisms by which Huazhuo Tiaozhi Granule (HTG) influences lipid metabolism, with a significant focus on the regulation of histone lactylation [78]. Additionally, the tRNA-derived fragment tRF-31R9J plays a crucial role in modulating the effects of tectorigenin (TEC) on ferroptosis by reducing histone lactylation and acetylation. This is achieved through the recruitment of HDAC1, which decreases these modifications and downregulates pro-ferroptosis genes such as ATF3, ATF4, and CHAC1. Consequently, this process inhibits hepatocyte ferroptosis and ameliorates MASH [79]. Overall, the exploration of lactylation in MASLD highlights the complexity of the disease and the potential for innovative diagnostic and therapeutic strategies.

### 4.2. Liver Fibrosis

Liver fibrosis is a pathological condition marked by the excessive accumulation of extracellular matrix (ECM) proteins, particularly fibrillar collagen, which can ultimately progress to cirrhosis and liver failure. This accumulation disrupts the normal liver architecture and function, leading to significant clinical complications. The pathogenesis of liver fibrosis involves a complex interplay of cellular and molecular mechanisms, including the activation of hepatic stellate cells (HSCs), which are the primary collagen-producing cells in the liver [80,81].

In the context of liver fibrosis, lactylation has been shown to regulate key processes such as HSCs activation and ECM deposition, which are central to fibrogenesis [74]. Recent studies have highlighted the role of HK2 in mediating gene expression through histone lactylation, which is essential for the activation of HSCs and the development of liver fibrosis. Inhibition of HK2 or lactate production can significantly reduce HSC activation and fibrosis, hinting that these pathways could be targeted for therapeutic purposes [82]. In another study, H3K18la was found to accelerate fibrosis by enhancing the transcription of SOX9, a pivotal regulator of HSC differentiation and ECM deposition [83]. Additionally, the m^6^A reader, insulin-like growth factor 2 mRNA binding protein 2 (IGF2BP2) has been identified as a regulator of glycolytic metabolism, indirectly facilitating histone lactylation and thereby amplifying HSC activation [84]. In the context of arsenite-induced liver fibrosis, histone lactylation boosts interferon-regulatory factor 4 (IRF4) expression by modifying H3K18la, promoting Th17 cell differentiation and IL-17A secretion, which activates HSCs. This sequence of events highlights the essential role of histone lactylation in modulating immune responses and fibrotic processes in the liver [85]. Interestingly, it has been demonstrated that liver fibrosis resulting from overtraining involves intricate biochemical pathways, particularly the lactylation of SH3 domain-containing 3 (SORBS3), causing its phase separation and interaction with flotillin 1. This process sorts F-box protein 2 (FBXO2) into small extracellular vesicles (sEVs), also known as lactate bodies. These lactate bodies are then released into circulation and subsequently induce hepatocyte apoptosis and HSCs activation, ultimately leading to liver fibrosis [86].

Beyond mechanistic insights, lactylation also exhibits clinical relevance. Specific lactylation signatures have been identified as classifiers for liver fibrosis phenotypes and predictors of HCC progression, offering potential for early diagnosis and risk stratification [87]. Additionally, dynamic lactylation modifications observed in cirrhosis patients undergoing umbilical cord mesenchymal stem cell (UC-MSC) therapy suggest its utility in monitoring therapeutic responses [88].

### 4.3. Liver Ischemia/Reperfusion Injury (IRI)

Recent studies have highlighted the critical role of lactylation in modulating inflammatory responses and cellular metabolism during IRI events [89]. In the context of liver IRI, lactylation has been implicated in the regulation of macrophage activity and inflammatory responses. For instance, hepatocyte heat shock protein A12A (HSPA12A) has been shown to inhibit macrophage chemotaxis and activation by suppressing glycolysis-mediated HMGB1 lactylation and secretion, thereby attenuating liver injury [90]. Specifically, lactate is found to prime KAT8, which in turn lactylates mitochondrial phosphoenolpyruvate carboxykinase 2 (PCK2) at K100. This modification enhances PCK2 kinase activity, leading to a cascade of metabolic changes that exacerbate ferroptosis during hepatic IRI [29].

### 4.4. Drug-Induced Liver Injury (DILI)

Emerging evidence highlighted the potential involvement of lactylation in the pathogenesis of DILI, a major cause of acute liver failure and a significant concern in clinical settings [91]. In the context of acetaminophen-induced liver injury, the absence of PGC-1α leads to increased lactylation of mitochondrial proteins, which can disrupt normal mitochondrial function and contribute to the pathogenesis of liver injury [92]. Further investigations have revealed that lactate inhibits the ubiquitination of caspase-11 by reducing its interaction with neuronal precursor cell-expressed developmentally downregulated 4 (NEDD4). This inhibition is facilitated by the lactylation of NEDD4, specifically at the K33 residue, which disrupts the protein interactions between caspase-11 and NEDD4. Consequently, this leads to an increase in non-canonical pyroptosis in macrophages, thereby worsening liver injury [93]. Further research is needed to fully elucidate the functional implications of lactylation in DILI and to explore its potential as a therapeutic target.

### 4.5. Liver Cancer

#### 4.5.1. Metabolic Adaptation

HCC is a significant public health concern, given that it is one of the most typical forms of primary liver cancer and a leading factor in cancer-related mortality globally [94]. A hallmark of cancer is metabolic reprogramming, which helps tumor cells satisfy the needs for rapid proliferation and survival. Targeting cancer metabolism represents a promising therapeutic approach. However, challenges such as metabolic plasticity and the necessity for combination therapies remain [95].

Recent research has demonstrated that lactylation modulates enzymes integral to key metabolic pathways, including the TCA cycle, and the metabolism of carbohydrates, amino acids, fatty acids, and nucleotides. For example, lactylation at lysine 28 can inhibit the activity of enzymes such as adenylate kinase 2, thereby promoting the proliferation and metastasis of HCC cells [96,97]. Additionally, lactylation can impact lipid metabolic processes, which are essential for the survival and proliferation of cancer cells. The epigenetic regulation of long noncoding RNA NEAT1 by the m6A reader YTH domain-containing family protein 1 (YTHDC1), influenced by histone lactylation, activates lipid metabolic pathways, thereby facilitating HCC progression [98]. However, the delactylation of programmed death-ligand 1 (PD-L1) at K189, mediated by HDAC2, facilitates its nuclear translocation, which significantly contributes to the acceleration of liver cancer growth both in vitro and in vivo by enhancing cholesterol production. This highlights the dual role of PD-L1 in not only modulating immune responses, but also in driving metabolic pathways that support tumor growth [99]. Moreover, the lactylation of IGF2BP3 is critical for the capture and stabilization of mRNAs involved in serine metabolism, such as PCK2 and nuclear factor erythroid 2-related factor 2 (NRF2). This stabilization enhances the expression of these genes, leading to the reprogramming of serine metabolism and the strengthening of the antioxidant defense system, collectively contributing to HCC progression [61]. Overall, lactylation emerges as a key player in HCC, influencing metabolic adaptation through multiple mechanisms. These findings not only deepen our understanding of the role of lactylation in HCC, but also suggest potential therapeutic targets for HCC treatment by targeting lactylation-related metabolic pathways.

#### 4.5.2. Cell Cycle, Proliferation, and Survival

Lactylation has emerged as a critical regulator of cellular processes central to carcinogenesis, particularly cell cycle progression [97,100,101,102]. In HCC, cyclin E2 lactylation has been shown to promote tumor cell growth. Notably, SIRT3 activation counteracts this effect by inducing apoptosis and suppressing in vivo tumorigenesis [103]. Similarly, targeting protein for Xklp2 (TPX2) lactylation drives HCC progression by disrupting its interaction with protein phosphatase 1, which enhances AURKA phosphorylation at T288 and accelerates cell cycle progression [104].

Emerging evidence has revealed that H3K56 lactylation-mediated oncogene expression facilitates HCC progression [56]. Beyond histones, lactylation of key regulatory proteins activates pro-tumorigenic pathways. For example, the lactylation of ABCF1 at K430 promotes HCC progression by activating the KDM3A-H3K9me2-HIF1A axis, highlighting the diverse roles of lactylation in modulating transcriptional networks within cancer cells [64]. Similarly, the lactylation of centromere protein A (CENPA) at K124 has been shown to significantly enhance its transcriptional activation ability. This modification facilitates the progression of HCC by promoting the expression of genes that drive tumor growth and proliferation [105]. Notably, yes-associated protein (YAP), an effector of the Hippo signaling pathway, undergoes K90 lactylation, which disrupts its interaction with exportin CRM1. Consequently, YAP becomes sequestered within the nucleus, thereby enhancing the malignancy of HCC [106]. In another study, lactylation at K102 facilitates YAP activation by preventing Ser127 phosphorylation, thereby enhancing its role in promoting malignant progression [107]. Collectively, these studies not only establish YAP as a critical oncogenic target of lactylation, but also delineate how lactylation subverts Hippo pathway regulatory control through multi-site, multi-mechanistic synergy. This reveals a precise molecular blueprint driving the malignant progression of HCC.

In addition to HCC, intrahepatic cholangiocarcinoma (iCCA) represents a rare and aggressive variant of liver cancer that originates from the epithelial cells of the intrahepatic bile ducts. It is recognized as the second most prevalent type of primary liver cancer, following HCC [108]. A recent study highlights that nucleolin lactylation at K477, driven by the acyltransferase P300 during increased glycolysis, promotes iCCA progression. This modification boosts the translation of MAP kinase-activating death domain protein (MADD) by preventing alternative splicing, thus enhancing iCCA cell growth and invasion via the MAPK pathway [109]. Overall, these findings highlight the central role of lactylation in hepatic malignancies through modulating cell cycle progression, oncogene expression, and transcriptional activation, which represents a promising target for therapeutic intervention.

#### 4.5.3. Invasion and Metastasis

Recently, lactylation has emerged as a significant regulator in the invasion and metastasis of HCC. One study highlights that lactate accumulation in the TME can drive the epithelial–mesenchymal transition (EMT) in liver cancer cells through the lactylation of twist family BHLH transcription factor 1 (TWIST1), a transcription factor critical for EMT. This modification enhances its nuclear import and transcriptional activity, promoting the acquisition of mesenchymal-like phenotypes and facilitating cancer cell invasion and metastasis [110]. Another investigation into the effects of lactylation on HCC metastasis focuses on Rab7A, a protein involved in multivesicular body (MVB) trafficking. Lactate-induced lactylation of Rab7A inhibits its GTPase activity, promoting MVB docking with the plasma membrane and facilitating tumor-derived exosome biogenesis. These exosomes, rich in proteins that alter the ECM, support pre-metastatic niche formation and enhance metastatic potential [16]. Furthermore, the lactylation of H3K18 has been shown to enhance the transcriptional activity of cysteine desulfurase (NFS1), a key enzyme in iron-sulfur cluster biosynthesis. This modification reduces the susceptibility of HCC cells to ferroptosis, thereby promoting metastasis following sublethal heat stress [111]. Additionally, the modification of insulin receptor substrate 1 (IRS1) by lactylation enhances its stability and activity, thereby promoting downstream signaling pathways that facilitate cancer cell proliferation and metastasis [112]. Collectively, these studies underscore the multifaceted role of lactylation in regulating invasion and metastasis in HCC, offering new insights into potential therapeutic strategies targeting this modification to combat liver cancer progression.

#### 4.5.4. Immune Evasion

Immune evasion is a critical factor in the progression and treatment resistance of HCC. The TME of HCC is characterized by a complex interplay of immune cells, signaling pathways, and molecular mechanisms that collectively contribute to immune suppression and tumor progression. One of the key features of HCC is its ability to evade immune surveillance, which is facilitated by various mechanisms, including the expression of immune checkpoint molecules such as PD-L1, which inhibits T-cell activity and promotes immune escape [113,114,115].

Lactylation has emerged as a significant player in the reprogramming of the TME and enables immune evasion [116,117,118,119,120,121]. In HCC, lactylation is crucial for modulating immune checkpoints like PD-L1, aiding tumor immune evasion. Protein arginine methyltransferase 3 (PRMT3) contributes to this by activating pyruvate dehydrogenase kinase 1 (PDHK1)-regulated glycolysis and enhancing the direct association of H3K18la with the PD-L1 promoter, highlighting the complex connection between metabolism and immune regulation [122]. Recently, serine- and arginine-rich splicing factor 10 (SRSF10) is gaining attention in HCC immunotherapy for its role in immune evasion. It regulates lactate production, creating a feedback loop with glycolysis and histone lactylation, which promotes M2 macrophage polarization and suppresses CD8^+^ T cell activity. This immunosuppressive environment challenges the effectiveness of immune checkpoint inhibitors like anti-PD-1 therapy [123]. Nuclear protein 1 (NUPR1) is highly expressed in tumor-associated macrophages (TAMs) and is crucial for immunosuppression and immunotherapy outcomes in HCC. Its upregulation promotes M2 macrophage polarization, increasing immune checkpoints like PD-L1 and signal regulatory protein alpha (SIRPA), leading to CD8^+^ T cell exhaustion and reduced immunotherapy response. Tumor-derived lactate boosts NUPR1 in macrophages via histone lactylation, creating a feedback loop that worsens immune suppression. This underscores the importance of targeting metabolic pathways to improve cancer therapy [124]. In another study, lactate was shown to enhance the stability and function of regulatory T cells (Tregs) by modulating MOESIN lactylation, which in turn enhances TGF-beta signaling pathways. This process is vital for maintaining the tumor’s immunosuppressive environment, aiding in immune evasion [125]. In HCC, junctional adhesion molecule-like protein (JAML) expression on CD8^+^ T cells is linked to better immunotherapy outcomes and prognosis. Notably, JAML^+^ CD8^+^ T cells correlate with LDHA^+^ CD4^+^ T cells, where LDHA facilitates the conversion of pyruvate to lactate, promoting CD4^+^ T cell differentiation into Th1 cells, crucial for activating CD8^+^ T cells in antitumor responses [126]. In summary, lactylation plays a crucial role in regulating immune escape in HCC by modulating immune checkpoint expression, altering metabolic pathways, and influencing the TME. Understanding these mechanisms provides valuable insights into potential therapeutic targets for enhancing the efficacy of immunotherapy in HCC.

#### 4.5.5. Therapy Resistance

Recent studies have highlighted the multifaceted role of lactylation in promoting resistance to various therapeutic agents used in HCC treatment. One of the key mechanisms by which lactylation contributes to therapy resistance is through the modulation of metabolic pathways. In lenvatinib-resistant HCC models, increased glycolysis leads to lactate accumulation, which in turn drives the lactylation of IGF2BP3. This boosts PCK2 and NRF2 expression, altering serine metabolism and enhancing antioxidant defenses, thus fostering lenvatinib resistance [61]. Similarly, lactylation of HECT domain E3 ubiquitin protein ligase 2 (HECTD2) has been shown to limit the response of HCC to lenvatinib by facilitating the degradation of Kelch-like ECH-associated protein 1 (KEAP1) and activating the KEAP1/NRF2 signaling pathway, which initiates an antioxidative response [127]. Furthermore, lactylation-driven ubiquitin-specific protease 34 (USP34) has been implicated in cisplatin resistance in HCC, where it regulates histone lactylation levels and promotes drug resistance [128]. Another study found that histone lactylation regulates the ubiquitin E3 ligase NEDD4, which in turn ubiquitinates and degrades PTEN. This downregulation of PTEN boosts glycolysis, increasing lactate production and histone lactylation, thereby sustaining drug resistance [129]. Specifically, the impact of lactylation extends to the TME, where it contributes to immunotherapy resistance. Mechanically, lactylation shapes the tumor immunosuppressive microenvironment by stimulating gene transcription within chromatin, thus promoting tumor progression and diminishing the efficacy of therapeutic agents [19].

In addition to drug resistance, histone lactylation also plays a crucial role in maintaining the stemness and radio-resistance of cancer stem cells (CSCs) in HCC. For instance, histone lactylation facilitates the expression of minichromosome maintenance complex component 7 (MCM7), a gene associated with CSC properties, thereby enhancing radio-resistance in HCC [130].

#### 4.5.6. Cancer Stemness

Lactate has garnered increasing recognition for its role in modulating cancer stemness and cellular plasticity through epigenetic mechanisms [131]. One study underscores the significance of histone metabolic and signaling pathways. Specifically, the lactylation of certain H3K56 and aldolase A (ALDOA) at K230/322 has been linked to the preservation of stemness characteristics in liver cancer stem cells (LCSCs), thereby contributing to the phenotypic and functional heterogeneity observed in HCC [132]. Furthermore, demethylzeylasteral, a triterpene anti-tumor compound, has been demonstrated to significantly reduce histone lactylation levels, thereby impairing the stemness and tumorigenic potential of LCSCs, offering a novel approach to targeting CSCs and their associated tumorigenic properties [133].

#### 4.5.7. Angiogenesis

Angiogenesis plays a crucial role in the progression and treatment of HCC. Anti-angiogenic therapies have improved the prognosis of HCC by targeting these pathways, although the survival benefits conferred by these therapies are modest, necessitating the identification of new targets [134,135].

It has been reported that lactate can influence the expression of genes involved in angiogenesis through histone lactylation [136]. One study demonstrated that increased lactylation of histones, especially at H3K9 and H3K18, occurs in endothelial cells during pathological angiogenesis, enhancing the transcription of angiogenic genes and promoting neovascularization in HCC [137]. Moreover, the role of lactate in angiogenesis is further supported by its ability to influence TAMs. These immune cells, when exposed to high lactate levels, can adopt a pro-angiogenic phenotype, further contributing to the neovascularization process within tumors [138]. In addition to its direct effects on endothelial cells and TAMs, lactate also impacts the expression of hypoxia-inducible factors (HIFs), which are crucial regulators of angiogenesis under hypoxic conditions. The stabilization and activation of HIFs in response to lactate accumulation can lead to increased expression of angiogenic factors such as VEGF, thereby promoting angiogenesis in HCC [64,139]. Golgi phosphoprotein 73 (GP73), when activated by histone lactylation and c-Myc, has also been reported to exert pro-angiogenic roles in HCC [140].

Overall, these studies underscore the multifaceted role of lactylation in HCC, influencing various aspects of tumor biology and offering potential avenues for therapeutic intervention. By targeting lactylation pathways, such as Royal jelly acid, it may be possible to develop novel strategies to improve treatment outcomes in HCC patients [141].

## 5. Therapeutic Strategies Targeting of Lactate and Lactylation in Liver Diseases

### 5.1. Targeting Lactate Availability/Production

In recent years, targeting lactate production and transport has emerged as a promising strategy to overcome drug resistance and enhance the efficacy of cancer treatments [1,142]. Inhibitors of lactate dehydrogenase (LDH), pyruvate dehydrogenase (PDH), and monocarboxylate transporters (MCTs) are being explored as therapeutic strategies to disrupt lactate homeostasis in tumors. These approaches aim to reduce lactate levels in the TME, thereby restoring immune function and sensitizing tumors to conventional therapies [2,143].

In the context of liver disease, oxamate, an LDHA inhibitor, has been reported to inhibit the differentiation of Th17 cells and HSCs, thereby mitigating hepatic fibrosis [82,85]. Under aerobic conditions, pyruvate is typically converted to acetyl-CoA through the action of the PDH complex. However, the application of rotenone, a PDH inhibitor, impedes this conversion, redirecting pyruvate metabolism towards lactate production and consequently facilitating lactylation [130,144]. Moreover, dichloroacetate involves the inhibition of pyruvate dehydrogenase kinase (PDHK), which leads to the activation of the PDH complex. This activation promotes the conversion of pyruvate to acetyl-CoA, thereby facilitating its entry into the TCA cycle for oxidative phosphorylation rather than conversion to lactate. This treatment has been demonstrated to inactivate HSCs and improve liver fibrosis [82]. Another promising strategy involves the use of 2-deoxy-D-glucose (2-DG), an HK inhibitor that interferes with glycolysis, potentially offering a new avenue to treat liver fibrosis or HCC [61,84,130].

MCTs, particularly MCT1 and MCT4, are instrumental in facilitating the transport of lactate across cellular membranes, thereby playing a crucial role in sustaining the glycolytic phenotype characteristic of cancer cells. Targeting these transporters can disrupt lactate efflux, resulting in intracellular lactate accumulation and subsequent metabolic stress [145,146]. Recent investigations have demonstrated that the MCT1 inhibitor AR-C155858 diminishes tumor growth by downregulating Tregs and upregulating the expression of anti-tumor cytokines [147]. Beyond MCT1, recent research has demonstrated that targeting MCT4 can disrupt the acidic TME, thereby mitigating the immunosuppressive conditions that impede effective T cell infiltration and activation. For example, the application of VB124, a potent MCT4 inhibitor, has been shown to suppress HCC tumor growth in immunocompetent mouse models by enhancing CD8^+^ T cell infiltration and cytotoxicity. Furthermore, the combination of MCT4 inhibition with immune checkpoint blockade (ICB) therapies, such as anti-PD-1, has been found to further improve therapeutic outcomes [148]. Moreover, MSC-4381, an MCT4 inhibitor, has been reported to suppress the proliferation of HCC, while simultaneously enhancing T cell cytotoxicity and reducing exhaustion [149]. Specifically, Syrosingopine, known primarily as an anti-hypertensive drug, has been identified as an inhibitor of lactate transporters MCT1 and MCT4. Recent studies have highlighted its role in the activation of HSCs, which are pivotal in the development of liver fibrosis [150]. Taken together, the strategic inhibition of MCTs represents a novel and promising avenue for therapeutic intervention in liver disease by modulating lactate availability and metabolic adaptations.

CD147, or Basigin, is a transmembrane glycoprotein crucial for regulating MCT1 and MCT4, which transport lactate and monocarboxylates across the plasma membrane. This process is vital for cellular metabolism and pH balance, especially in tumor cells that rely on glycolysis for energy. CD147 ensures the proper placement and function of MCTs on the cell surface [151]. In a recent study, CD147 has been shown to contribute to the reprogramming of glucose metabolism in HCC cells through a p53-dependent mechanism. Inhibition of CD147 and/or MCT1 can suppress the proliferation of HCC cells by downregulating glucose metabolism [152].

Lysyl oxidase (LOX) is another significant metabolic modulator implicated in liver disease. A promising therapeutic strategy involves the deployment of an injectable nanoparticle–hydrogel composite system engineered to deplete intratumoral lactate, a key contributor to the immunosuppressive TME. By reducing lactate levels via LOX, this system aims to convert the TME from an immunosuppressive to an immunocompetent state, thereby augmenting the efficacy of immunotherapy against residual HCC cells [153]. Another study presents an innovative approach utilizing a platelet membrane-engineered nanoparticle loaded with erastin, superparamagnetic iron oxide (SPIO) nanoparticles, and LOX to enhance HCC treatment through ferroptosis and immunotherapy. The removal of lactic acid generates hydrogen peroxide, which subsequently reacts with Fe^2+^/Fe^3+^ released from SPIO to produce cytotoxic hydroxyl radicals, thereby enhancing ferroptosis and chemodynamic therapy [154] (Table 1).

### 5.2. Inhibiting Lactylation “Writer” Activity

The dynamic nature of lactylation is governed by specific enzymes known as “writer”, “reader”, and “eraser”, which add, recognize, and remove lactyl groups, respectively. Inhibiting the activity of lactylation “writers”, the enzymes responsible for this modification, may be crucial in the management of liver diseases. For instance, AARS1 has been identified as a lactate sensor and lactyltransferase, playing a significant role in the lactylation of p53, a crucial tumor suppressor protein. It mediates global Kla in tumor cells by binding to lactate and catalyzing the formation of lactate-AMP, which is then transferred to lysine residues on target proteins. β-alanine, a non-essential amino acid, has been shown to interfere with the binding of lactate to AARS1. This disruption leads to a reduction in p53 lactylation, thereby affecting its tumor suppressor functions [38]. Furthermore, the inhibition of KAT8 with KAT8-IN-1 or MC4033 could potentially serve as a therapeutic strategy to mitigate ferroptosis and protect against liver damage during IRI [29].

### 5.3. Modulating Lactylation “Eraser” Activity

HDAC1-3 and the Sirtuin family proteins have been recognized as effective mediators in the removal of Kla [35,54,155]. Recent studies show that HDAC inhibitors like apicidin and MS275 significantly affect histone modifications essential for gene expression and cell function. They upregulate H3K18 acetylation, which competes with and downregulates H3K18la, inactivating HSCs, crucial in liver fibrosis progression [82]. Conversely, the delactylation of cyclin E2 by the SIRT3 activator Honokiol suppresses its oncogenic activity, thus hindering the advancement of HCC [103]. The diverse functions of HDACs and SIRTs in disease progression may be attributed to their interactions with other molecular modifications.

### 5.4. Combinatorial Inhibition of Lactate Availability and Lactylation

**Figure 4:**
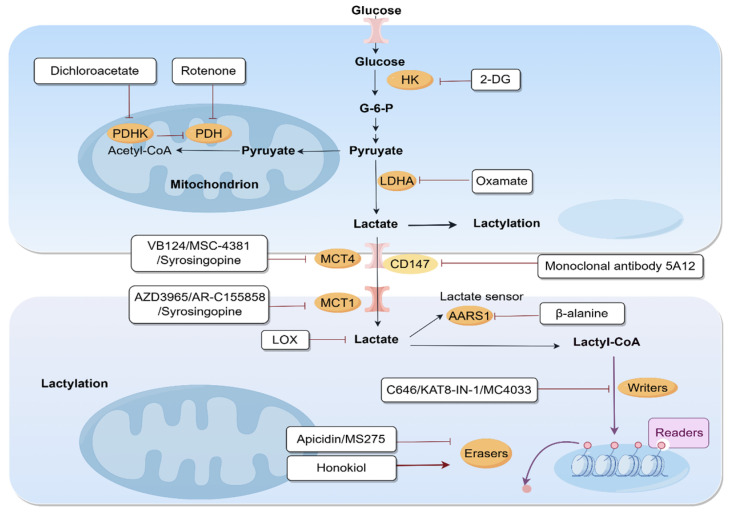
Therapeutic targeting of the lactate–lactylation axis in liver diseases. This schematic illustrates potential therapeutic strategies targeting key steps in lactate production, transport, and lactylation signaling. AARS1, alanyl-tRNA synthetase 1; 2-DG, 2-deoxy-D-glucose; G-6-P, glucose-6-phosphate; HK, hexokinase; LDHA, lactate dehydrogenase A; LOX, lysyl oxidase; MCT1, monocarboxylate transporter 1; MCT4, monocarboxylate transporter 4; PDH, pyruvate dehydrogenase; PDHK, pyruvate dehydrogenase kinase.

The combinatorial inhibition of lactate availability and lactylation presents a promising therapeutic avenue in liver disease. Recent research has indicated that the dual inhibition of MCT1 in conjunction with P300 inhibitors could potentially reduce exosome-mediated metastasis in HCC [16]. Collectively, these insights into the role of lactate and lactylation in disease mechanisms underscore the importance of maintaining lactate homeostasis and modulating lactylation, and offer promising avenues for targeted therapies in liver diseases (Figure 4).

### 5.5. Potential Risks of Lactylation-Targeted Therapies

Although therapies targeting lactylation demonstrate significant therapeutic potential, recent evidence indicates that their efficacy and safety may be context-dependent. Intriguingly, HDAC inhibitors appear to inactivate HSCs, sensitize cancer cells to ferroptosis, and enhance the sensitivity of cancer cells to cisplatin therapy, while SIRT3 activators suppress HCC growth [103,156,157]. Future research should aim to elucidate the context-dependent effects of lactylation and develop strategies to selectively modulate its activity, thereby maximizing therapeutic benefits while minimizing potential risks. Consequently, forthcoming therapies should prioritize cell-type-specific delivery, stage-adapted dosing, and biomarker-guided patient stratification.

In conclusion, the emerging understanding of lactate and lactylation in HCC provides a compelling rationale for developing lactylation-directed therapeutics among liver diseases. By targeting the metabolic and epigenetic modifications driven by lactate, it is possible to overcome the immunosuppressive barriers and therapy resistance that characterize HCC. This approach not only holds promise for improving the efficacy of existing treatments, but also offers a pathway to novel therapeutic strategies that could significantly impact patient outcomes in HCC.

## 6. Challenges and Future Directions

The discovery of lactylation has greatly enhanced the understanding of its role in liver diseases. However, challenges remain in clarifying its mechanisms and applying this knowledge clinically. Understanding the enzymes that add and remove lactyl groups is important for grasping this PTM’s dynamics. Indeed, lactylation is a reversible modification regulated by dedicated “eraser” enzymes such as HDAC1-3, SIRT1-3, and CobB. This reversibility positions lactylation as a dynamic metabolic sensor akin to acetylation [35,36]. However, research on “reader” proteins that recognize lactylation and trigger effects is incomplete. Additionally, the downstream signaling pathways associated with these modifications are not well characterized. The precise mechanisms by which lactylation at specific lysine residues modulates protein activity, stability, localization, or interactions, thereby influencing cellular fate and pathological processes, are not yet fully understood.

Although direct studies focusing on liver zonation remain limited, emerging evidence indicates the presence of contextual plasticity. Specifically, pericentral hepatocytes demonstrate an increased glycolytic flux and elevated lactate production [12], implying the existence of zone-specific lactylation patterns. In the future, researchers need to map lactylation dynamics across liver zones using spatial-omics in acute or chronic liver disease, so as to provide effective methods for prevention, diagnosis, and treatment of related liver diseases. Furthermore, comparative analyses between lactylation and other metabolite-driven PTMs (e.g., crotonylation, succinylation, β-hydroxybutyrylation) may reveal unique or overlapping pathogenic contributions in liver microenvironments, warranting dedicated investigation.

Alterations in lactylation sites or levels may offer potential for disease diagnosis, prognosis, or efficacy prediction. Among these, H3K18la has emerged as a significant marker of tissue-specific active enhancers, suggesting its involvement in the regulation of genes critical for tissue-specific functions [158]. In terms of clinical translation, ongoing clinical trials are investigating the correlation between H3K18la in serum monocytes and postoperative delirium and its severity in patients undergoing cardiovascular surgery (NCT06007755). Nonetheless, the specificity, sensitivity, stability, and detectability of these modifications in bodily fluids such as blood and feces necessitate validation through extensive clinical studies. Moreover, there is a notable deficiency in targeted intervention strategies in human cohorts. The development of safe and effective modulators, including targeting lactate availability/transport and lactylation, presents significant challenges. Additionally, there is increasing interest in exploring rational and synergistic strategies for drug combinations. The goal of these combination therapies is to address multiple pathways simultaneously, which decreases the chance of drug resistance and boosts overall treatment effectiveness.

## 7. Conclusions

In summary, while lactylation-targeted therapies show promise for treating liver diseases, they are still in early development and need further research. Lactylation acts as a metabolic checkpoint, linking environmental signals to disease progression in these areas. To develop more effective therapeutic strategies, it is crucial to thoroughly understand how lactate interacts with different metabolic and epigenetic processes in these diseases. By shedding light on these complex associations, researchers can formulate new therapies that take advantage of lactate’s unique properties. Future studies should focus on clinically validating these strategies, particularly for patients with metabolic comorbidities.

## Figures and Tables

**Table 1:** Preclinical evidence of therapeutic strategies targeting lactate and lactylation in liver disease.

Target	Metabolic Modulators or Drugs	Biological Processes	Role	References
LDHA	Oxamate	Liver fibrosis	suppress Th17 cell differentiation and inactivate HSCs	[82,85]
PDH	Rotenone	HCC	lactylation, facilitate MCM7 expression	[130,144]
PDHK	Dichloroacetate	Liver fibrosis	inhibit HSCs	[82]
HK	2-DG	Liver fibrosis	ameliorate liver fibrosis	[84]
HK	2-DG	HCC	inhibit lactylation, reduce MCM7 expression	[130]
HK	2-DG; combine with lenvatinib	HCC	increase lenvatinib sensitivity and reduce IGF2BP3 lactylation	[61]
MCT1	AR-C155858; combine with anti-PD-1	HCC	downregulate Tregs and upregulating anti-tumor cytokine expression	[147]
MCT4	VB124; combine with an-ti-PD-1	HCC	enhance CD8^+^ T cell infiltration and cytotoxicity	[148]
MCT4	MSC-4381	HCC	enhance T cell cytotoxicity and reducing exhaustion	[149]
MCT1/4	Syrosingopine	Liver fibrosis	activate HSCs and exacerbate liver fibrosis	[150]
CD147	Monoclonal antibody 5A12	HCC	suppress the proliferation of HCC cells	[152]
MCT1	AR-C155858	HCC	suppress the proliferation of HCC cells	[152]
Lactate	LOX; combine with immunotherapy	HCC	inhibit residual HCC growth and lung metastasis	[153]
Lactate	LOX; combine with immunotherapy	HCC	enhance ferroptosis in tumors	[154]
AARS1	β-alanine	Cancer	reduce p53 lactylation, and mitigates tumorigenesis	[38]
KAT8	KAT8-IN-1; MC4033	IRI	suppress hyperlactatemia-mediated hepatic IRI and ferroptosis	[29]
Class I HDAC	Apicidin and MS275	Liver fibrosis	inactivate HSCs	[82]
SIRT3	Honokiol	HCC	induce HCC cell apoptosis and prevent HCC outgrowth	[103]
MCT1 and P300	AZD3965 and C646	HCC	abrogate HCC metastasis	[16]

AARS1, alanyl-tRNA synthetase 1; 2-DG, 2-deoxy-D-glucose; HCC, hepatocellular carcinoma; HDAC, histone deacetylase; HK, hexokinase; HSCs, hepatic stellate cells; IRI, ischemia/reperfusion injury; KAT8, lysine acetyltransferase 8; LDHA, lactate dehydrogenase A; LOX, lysyl oxidase; MCM7, minichromosome maintenance complex component 7; MCT1, monocarboxylate transporter 1; MCT4, monocarboxylate transporter 4; PDH, pyruvate dehydrogenase; PDHK, pyruvate dehydrogenase kinase; SIRT3, sirtuin 3.

## Data Availability

No new data were created or analyzed in this study.

## Abbreviations

AARS1/2	alanyl-tRNA synthetase 1/2
ABCF1	ATP-binding cassette subfamily F member 1
ACSS2	acetyl-CoA synthetase 2
ALDOA	aldolase A
ATAT1	α-tubulin acetyltransferase 1
BMP2	bone morphogenetic protein 2
CENPA	centromere protein A
CSCs	cancer stem cells
2-DG	2-deoxy-D-glucose
DILI	drug-induced liver injury
ECM	extracellular matrix
EMT	epithelial-mesenchymal transition
FASN	fatty acid synthase
FBXO2	F-box protein 2
G3P	glyceraldehyde-3-phosphate
GCN5	general control non-depressible 5
GLO1	glyoxalase 1
GLO2	glyoxalase 2
GP73	Golgi phosphoprotein 73
GTPSCS	guanosine triphosphate-specific succinyl-CoA synthetase
HATs	histone acetyltransferases
H3K18la	histone H3 lysine 18 lactylation
HCC	hepatocellular carcinoma
HDACs	histone deacetylases
HIF1A	hypoxia-inducible factor 1A
HIFs	hypoxia-inducible factors
HK2	hexokinase 2
HMGB1	high mobility group box-1
HSCs	hepatic stellate cells
HSPA12A	hepatocyte heat shock protein A12A
HTG	Huazhuo Tiaozhi Granule
ICB	immune checkpoint blockade
iCCA	intrahepatic cholangiocarcinoma
IGF2BP2	insulin-like growth factor 2 mRNA binding protein 2
IRF4	interferon-regulatory factor 4
IRI	ischemia/reperfusion injury
IRS1	insulin receptor substrate 1
JAML	junctional adhesion molecule-like protein
KAT	lysine acetyltransferase
KDM3A	lysine demethylase 3A
KEAP1	Kelch-like ECH-associated protein 1
Kla	lysine lactylation
LCSCs	liver cancer stem cells
LDH	lactate dehydrogenase
LDHA	lactate dehydrogenase A
LGSH	lactoylglutathione
L-La CoA	L-lactyl-CoA
LOX	lysyl oxidase
m6A	N6-methyladenosine
MADD	MAP kinase-activating death domain protein
MASH	metabolic dysfunction-associated steatohepatitis
MASLD	metabolic dysfunction-associated steatotic liver disease
MCM7	minichromosome maintenance complex component 7
MCTs	monocarboxylate transporters
METTL3	methyltransferase-like 3
MGO	methylglyoxal
MVB	multivesicular body
NAFLD	non-alcoholic fatty liver disease
NAM	nicotinamide
NEDD4	neuronal precursor cell-expressed developmentally downregulated 4
NRF2	nuclear factor erythroid 2-related factor 2
NFS1	cysteine desulfurase
NUPR1	nuclear protein 1
PCK2	phosphoenolpyruvate carboxykinase 2
PDC	pyruvate dehydrogenase complex
PDH	pyruvate dehydrogenase
PDHX	pyruvate dehydrogenase complex component X
PDHK	pyruvate dehydrogenase kinase
PD-L1	programmed death-ligand 1
PRMT3	protein arginine methyltransferase 3
PTM	post-translational modification
sEVs	small extracellular vesicles
SIRPA	signal regulatory protein alpha
SORBS3	SH3 domain-containing 3
SPIO	superparamagnetic iron oxide
SRSF10	serine- and arginine-rich splicing factor 10
TAMs	tumor-associated macrophages
TCA	tricarboxylic acid
TEC	tectorigenin
TME	tumor microenvironment
TPX2	targeting protein for Xklp2
Tregs	regulatory T cells
TWIST1	twist family BHLH transcription factor 1
UC-MSC	umbilical cord mesenchymal stem cell
USP34	ubiquitin-specific protease 34
YAP	yes-associated protein
YTHDC1	YTH domain-containing family protein 1
